# Language Models for Multilabel Document Classification of Surgical Concepts in Exploratory Laparotomy Operative Notes: Algorithm Development Study

**DOI:** 10.2196/71176

**Published:** 2025-07-09

**Authors:** Jeremy A Balch, Sasank S Desaraju, Victoria J Nolan, Divya Vellanki, Timothy R Buchanan, Lindsey M Brinkley, Yordan Penev, Ahmet Bilgili, Aashay Patel, Corinne E Chatham, David M Vanderbilt, Rayon Uddin, Azra Bihorac, Philip Efron, Tyler J Loftus, Protiva Rahman, Benjamin Shickel

**Affiliations:** 1 Department of Surgery, University of Florida College of Medicine, Gainesville, FL, United States; 2 Department of Health Outcomes and Biomedical Informatics, University of Florida College of Medicine, Gainesville, FL, United States; 3 University of Florida College of Medicine, Gainesville, FL, United States; 4 Intelligent Clinical Care Center, University of Florida, Gainesville, FL, United States; 5 Department of Medicine, University of Florida College of Medicine, 1600 SW Archer Road, PO Box 100224, Gainesville, FL, 32610, United States, 3522739958, 1 3522739221

**Keywords:** chart review, generative large language models, general surgery, natural language processing, exploratory laparotomy

## Abstract

**Background:**

Operative notes are frequently mined for surgical concepts in clinical care, research, quality improvement, and billing, often requiring hours of manual extraction. These notes are typically analyzed at the document level to determine the presence or absence of specific procedures or findings (eg, whether a hand-sewn anastomosis was performed or contamination occurred). Extracting several binary classification labels simultaneously is a multilabel classification problem. Traditional natural language processing approaches—bag-of-words (BoW) and term frequency-inverse document frequency (tf-idf) with linear classifiers—have been used previously for this task but are now being augmented or replaced by large language models (LLMs). However, few studies have examined their utility in surgery.

**Objective:**

We developed and evaluated LLMs for the purpose of expediting data extraction from surgical notes.

**Methods:**

A total of 388 exploratory laparotomy notes from a single institution were annotated for 21 concepts related to intraoperative findings, intraoperative techniques, and closure techniques. Annotation consistency was measured using the Cohen κ statistic. Data were preprocessed to include only the description of the procedure. We compared the evolution of document classification technologies from BoW and tf-idf to encoder-only (Clinical-Longformer) and decoder-only (Llama 3) transformer models. Multilabel classification performance was evaluated with 5-fold cross-validation with *F*_1_-score and hamming loss (HL). We experimented with and without context. Errors were assessed by manual review. Code and implementation instructions may be found on GitHub.

**Results:**

The prevalence of labels ranged from 0.05 (colostomy, ileostomy, active bleed from named vessel) to 0.50 (running fascial closure). Llama 3.3 was the overall best-performing model (micro *F*_1_-score 0.88, 5-fold range: 0.88-0.89; HL 0.11, 5-fold range: 0.11-0.12). The BoW model (micro *F*_1_-score 0.68, 5-fold range: 0.64-0.71; HL 0.14, 5-fold range: 0.13-0.16) and Clinical-Longformer (micro *F*_1_-score 0.73, 5-fold range: 0.70-0.74; HL 0.11, 5-fold range: 0.10-0.12) had overall similar performance, with tf-idf models trailing (micro *F*_1_-score 0.57, 5-fold range: 0.55-0.59; HL 0.27, 5-fold range: 0.25-0.29). *F*_1_-scores varied across concepts in the Llama model, ranging from 0.30 (5-fold range: 0.23-0.39) for class III contamination to 0.92 (5-fold range: 0.98-0.84) for bowel resection. Context enhanced Llama’s performance, adding an average of 0.16 improvement to the *F*_1_-scores. Error analysis demonstrated semantic nuances and edge cases within operative notes, particularly when patients had references to prior operations in their operative notes or simultaneous operations with other surgical services.

**Conclusions:**

Off-the-shelf autoregressive LLMs outperformed fined-tuned, encoder-only transformers and traditional natural language processing techniques in classifying operative notes. Multilabel classification with LLMs may streamline retrospective reviews in surgery, though further refinements are required prior to reliable use in research and quality improvement.

## Introduction

Operative notes represent the most thorough narrative of a surgical case in the electronic health record, containing information that is largely inaccessible outside of manual human review [1,2]. This limitation impedes retrospective studies on surgical technique and intraoperative findings that impact outcomes, as well as the ability to perform prospective validation and real-time implementation of decision-support systems. Natural language processing (NLP) and large language models (LLMs) may offer a streamlined approach to information extraction for clinical workflow, education, research, performance improvement, and billing purposes [3].

The terms and phrases used to characterize surgical techniques and intraoperative findings often contain complex dependencies that span multiple sentences and are best understood in the context of an entire operative note. Furthermore, in retrospective reviews focused on patient outcomes, operative notes serve as a vehicle to identify study participants, with subsequent attention to downstream outcomes often represented in structured data (ie, mortality, surgical site infection, or anastomotic leaks defined by the *International Classification of Disease* codes) [4,5].

For this reason, we frame our problem in terms of a multilabel document classification task [6] where operative notes take on a series of binary labels as to whether or not a certain intraoperative finding (eg, bleeding and contamination) or technique (eg, bowel resection, hand-sewn anastomosis, and style of fascial closure) occurred during the case. Traditional NLP methods, using word frequencies, generally perform well on this task, though can fail to capture context and negation, a noted strength of the attention mechanism in LLMs [7]. Several studies have investigated LLMs for text classification in clinical notes, though to our knowledge, few studies have examined multilabel classification, only one has used generative models, and none have done so in surgical specialties [8-12]. There is a similar paucity of publications using real-world data outside of curated datasets, which, in addition to representing idealized clinical documentation, are also conspicuously devoid of operative notes [13-15].

Generative LLMs may offer “off-the-shelf” abilities to capture the multidependency nature of intraoperative findings and surgical techniques. We hypothesize that generative LLMs can outperform fine-tuned encoder-only LLMs and traditional NLP methods in classifying operative notes as containing specific findings and techniques [16].

## Methods

### Data

Using the University of Florida Health Integrated Data Repository as an honest broker, we assessed 2 single-center, longitudinal electronic health record datasets for all adult patients admitted to a surgical service at University of Florida Health Gainesville and Jacksonville, both quaternary referral centers, between June 1, 2014, and August 22, 2022. We randomly selected 420 fully deidentified exploratory laparotomy operative reports using SQL queries. In total, 32 were found to be mislabeled as “exploratory laparotomy,” with no evidence that the abdominal cavity was entered, and so were excluded, leaving 388 notes. As our scope was limited to the operative notes themselves, no surgical outcome, operative metadata, or sociodemographic data were collected.

### Ethical Considerations

This study was approved by the University of Florida Institutional Review Board and Privacy Office (IRB#201600262) as an exempt study with a waiver of informed consent. All data used in this study were deidentified. No compensation was provided. This study was performed in accordance with the TRIPOD+LLM (Transparent Reporting of a Multivariable Prediction Model for Individual Prognosis or Diagnosis+Large Language Model) reporting guideline [17].

### Data Preparation

**Figure 1.**
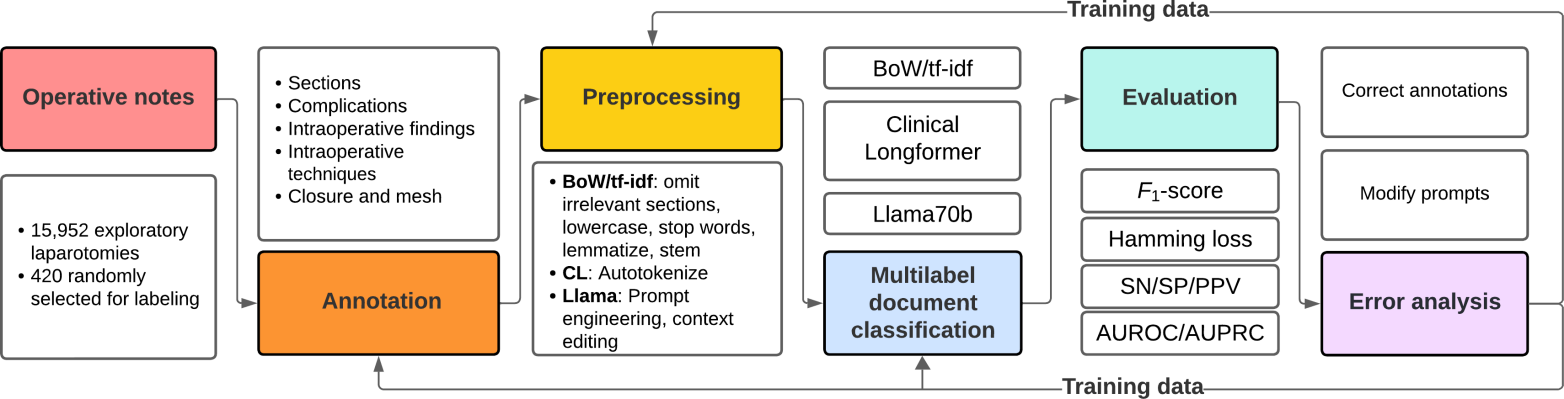
Workflow schema. Exploratory laparotomy notes are first extracted and annotated. After preprocessing, they are passed to 4 machine-learning models for multilabel document classification. Models are compared using several performance metrics. Finally, error analysis is performed and all annotation, preprocessing, prompts, and model architectures are modified as necessary on training data to optimize the *F*_1_-score prior to evaluation on test data. AUPRC: area under the precision-recall curve; AUROC: area under the receiver operating characteristic curve; BoW: bag-of-words; CL: Clinical-Longformer; PPV: positive-predictive value; SN: sensitivity; SP: specificity; tf-idf: term frequency-inverse document frequency.

The project workflow is shown in Figure 1. A team of 8 annotators, consisting of medical students (TRB, LMB, YP, A Bilgili, AP, CEC, RU, and DMV) and one surgical resident (JAB), were trained on the project’s objectives and annotation software. A detailed annotation manual is provided with definitions, categories, and illustrative examples (Multimedia Appendix 1). An annotated operative note is shown in Multimedia Appendix 2. Emphasis was placed on achieving a high level of consistency, with the goal of reaching a Cohen κ coefficient of above 0.8 for interrater reliability [18]. The first author (JAB) served as the ground truth. A total of 20 operative notes were set aside for annotator training and were reviewed by all annotators. Following training, annotators participated in regular discussions to address any challenges and were reviewed by the first author. Annotations were performed with Label Studio (version 1.8.2; HumanSignal).

### Labels

Notes were annotated for structure, intraoperative findings, and surgical techniques. Whole text spans were highlighted based on note structure: patient or staff or anesthesia personnel information; procedures performed; pre- and postoperative diagnoses; intraoperative findings; indication or history; description of the procedure; ins, outs, and specimens; disposition; and complications. Intraoperative findings included: contamination (class I, II, III, and IV) as defined in the peer-reviewed literature [19]; and bleeding, differentiating between active bleed from a named vessel and active bleed from a solid organ. Whole-document labels were performed for: bowel resection, primary repair of enterotomies, colostomy formation, ileostomy formation, hand-sewn anastomosis, stapled anastomosis, placement of mesh, fascia closure techniques (running or continuous, interrupted, and left open), and skin closure techniques (full, Prevena, partial, and left open). For the training set, Cohen κ across individual labels ranged from 0.39 to 1.0 with a mean and median agreement of 0.67 (SD 0.33) and 0.77 (IQR 0.52-1.0), respectively (Table S1 in Multimedia Appendix 3). The κ scores across all medical students are shown in Table S1 Multimedia Appendix 3. Because this was below our stated goal, additional training was provided with emphasis on these concepts, and each op note in the dataset was personally reviewed by the lead author. A total of 50 notes were annotated by each of the annotators, with the lead author annotating an additional 50.

### Data Splitting and Stratification for Class Imbalance

Standard techniques in multilabel classification tasks with label-specific class imbalances may result in datasets missing rare, positive labels [20,21]. To account for this, we performed iterative stratification from scikit-multilearn, splitting the data into 5-fold of training (80%) and test (20%) sets [20-22]. The distributions of the labels in each train and test set are shown in Table S2 in Multimedia Appendix 3.

Unlike other models, the Llama models were not fine-tuned on a hold-out training set. They were instead used to evaluate only the test set in each cross-validation fold.

### Models

We studied the traditional NLP multilabel document classification techniques with bag-of-words (BoW) and term frequency-inverse document frequency (tf-idf) approaches paired with logistic regression classifiers, as well as pretrained transformer models, the encoder-only Clinical-Longformer [23], and the decoder-only Llama herd (Llama 3.1 -3b, 8b, 70b, 3.2, and 3.3) [24].

BoW takes tokenized words and performs a classification task based on the frequency of the terms in a particular document. tf-idf applies a weight-based filter on the frequency of a term across a corpus of documents and evaluates the uniqueness of a word to a specific class.

Transformer-based models can leverage contextual information [25]. Encoder models process the entire document by systematically masking these tokens and predicting their values. While encoder models typically excel at classification tasks, their utility is often limited by length, as most models cannot process more than 512 tokens at a time [26]. Longformer models extend that range using both global and sliding-window attention mechanisms [27]. Li et al [23] fine-tuned a Clinical-Longformer model on clinical text from the Medical Information Mart for Intensive Care-III dataset [15] with a context of 4096 tokens, which outperformed Bidirectional Encoder Representations from Transformers (BERT) [28], ClinicalBERT [29], and BioBERT [30] on inference, question-answering, and classification tasks [23]. Finally, autoregressive decoder–only transformers estimate the probability distribution of the next token in a sequence based on the preceding tokens. As they are self-hosted, Llama allows for the secure handling of sensitive patient information and for this reason, these models were selected for this study [24]. The results shown below are the best-performing Llama 3.3 model.

### Preprocessing

Notes were reduced to the “description of procedure” as other parts of the note may contain information from previous procedures that may bias the model. For the tf-idf and BoW models, all texts were converted to lowercase, and common stop words (eg, “the,” “and,” and “is,”), punctuation, and numbers were removed. Stemming and lemmatization were performed to reduce words to their root forms (eg, “maturing” to “mature”). The text was then vectorized as combinations of unigrams, bigrams, trigrams, and 4-grams. We introduced padding to ensure that all sequences had a uniform length. The Clinical-Longformer and Llama models were tokenized using the Hugging Face autotokenizer [31].

### Model Hyperparameters

For BoW and tf-idf, we used logistic regression as our classifier. Hyperparameter search within each fold of the training data revealed marginally increased performance with L2-regularization strength of 0.1 and 10 for BoW and TFIDF overall, respectively. No other hyperparameters were modified based on the results of the test set. In the Clinical-Longformer model, we weighted the binary cross-entropy loss for each label inversely proportional to its prevalence in the training set given class imbalance. The model was optimized for the micro *F*_1_-score and trained for up to 500 epochs with early stopping, using a patience of 10 to prevent overfitting. The inference was run on an NVIDIA A100 8GB graphics processing unit in the University of Florida HiPerGator cluster. The Llama 3.3 model had the longest runtime, at 723 minutes.

A custom Python script was developed using the LlamaIndex framework for the Llama model [32]. Each task was a modified version of the annotation instructions, and the model was prompted with the operative note, the context of the task, few-shot instructions, a question, and a desired response format (Multimedia Appendix 4). A general context document was also provided and included brand names of mesh types, a description of types of skin closure, and other domain-specific knowledge that could aid in understanding patient notes and tasks (Multimedia Appendix 5). Given the 5-fold cross-validation design, all notes appeared in at least one test set. As a result, prompts were adjusted based on the model’s generated rationale for randomly selected errors on the whole dataset (eg, differentiating “primary repair” from “anastomosis” or clarifying the use of “prolene” in mesh vs suture contexts). Performance metrics were not evaluated during prompt tuning to avoid test set leakage.

### Model Evaluation

Overall performance was evaluated using the micro *F*_1_-score, which calculates the harmonic mean of precision and recall across all classes, and hamming loss (HL), which measures the fraction of misclassified labels relative to the total ground truth labels (with 0 indicating perfect classification). The mean and range of scores over 5 folds were reported. Optimal cutoffs were determined by maximizing the *F*_1_-score in 0.01 increments. Sensitivity, positive predictive value (PPV), specificity, area under the receiver operating curve, and area under the precision-recall curve were also reported. Individual label *F*_1_-scores were calculated using the “binary” average.

### Error Analysis

A total of 5 false positive and 5 false negative labels with the highest predicted probabilities were reviewed for each label using the best-performing Clinical-Longformer and Llama model. Several annotation errors were encountered during each iteration which resulted in manual reannotation by the lead author, repeat BoW, tf-idf, and Clinical-Longformer model training, and rerunning of the evaluation pipeline. The reported metrics reflect the latest training and evaluation.

### Data Availability and Code

Code and implementation instructions may be found on GitHub [33]. A toy dataset is provided using GPT-generated op notes and random labels.

## Results

### Data

Of the 388 operative notes, note length ranged from 73 to 1713 words, with a mean of 500 (SD 291) words and a median of 421 (IQR 292-603) words. Most notes were composed by the Trauma and Acute Care Surgery Department (n=267, 68.8%), with the remaining notes in Transplant Surgery (n=83, 21.4%) and Urology (n=30, 7.7%) along with combination cases with Vascular Surgery (n=24, 6.2%), Cardiothoracic Surgery (n=16, 4.1%), and Neurosurgery (n=8, 2.1%). We noted the class imbalance in the labels, as shown in Table 1.

**Table 1.** Prevalence of labels in the dataset.

Label		Prevalence
Intraoperative findings		
Active bleeding from the named vessel		0.05
Active bleeding from solid organ		0.11
Class I		0.34
Class II		0.48
Class III		0.16
Class IV		0.14
Intraoperative techniques		
Bowel resection		0.30
Primary repair		0.05
Serosal tear repair		0.05
Colostomy		0.12
Ileostomy		0.08
Hand-sewn anastomosis		0.12
Stapled anastomosis		0.15
Closure techniques		
Fascia closed (interrupted)		0.10
Fascia closed (running or continuous)		0.50
Fascia left open		0.32
Skin closed (full with Prevena)		0.04
Skin closed (full)		0.41
Skin closed (partial)		0.05
Skin left open		0.43
Synthetic		0.06

### Collective Performance Across All Labels

Overall mean micro *F*_1_-scores, along with minimum and maximum score per fold, are shown in Table 2. BoW (0.68, 5-fold range: 0.64‐0.71) outperformed tf-idf (0.57, 5-fold range: 0.55-0.59) overall with an increase in micro *F*_1_-score of 0.1 and a decrease in HL of two-fold. Comparing the encoder-only and decoder-only model architectures, Llama 3.3 (0.88, 5-fold range: 0.88-0.89) had generous improvement overall in the micro *F*_1_-score with equivalent HL to BoW and Clinical-Longformer.

We compared the Llama 3 series of models and observed a general trend of improved performance with increasing model size. An exception was Llama 3.2, which performed poorly—consistent with prior reports of its reduced effectiveness on medical datasets [34]. Results are presented in Figure S1 in Multimedia Appendix 6.

**Table 2.** Mean overall performance of models across all labels across all 5-folds^a^.

Model	Micro *F*_1_-score, mean (range)	Hamming loss, mean (range)
BoW^b^	0.68 (0.64-0.71)	0.14 (0.13-0.16)
tf-idf^c^	0.57 (0.55-0.59)	0.27 (0.25-0.29)
Clinical-Longformer	0.73 (0.70-0.74)	0.11 (0.10-0.12)
Llama 3.3	0.88 (0.88-0.89)	0.12 (0.11-0.12)

a Values in parentheses indicate the minimum and maximum performance.

b BoW: bag-of-words.

c tf-idf: term frequency-inverse document frequency.

### Individual Label Performance

**Figure 2.**
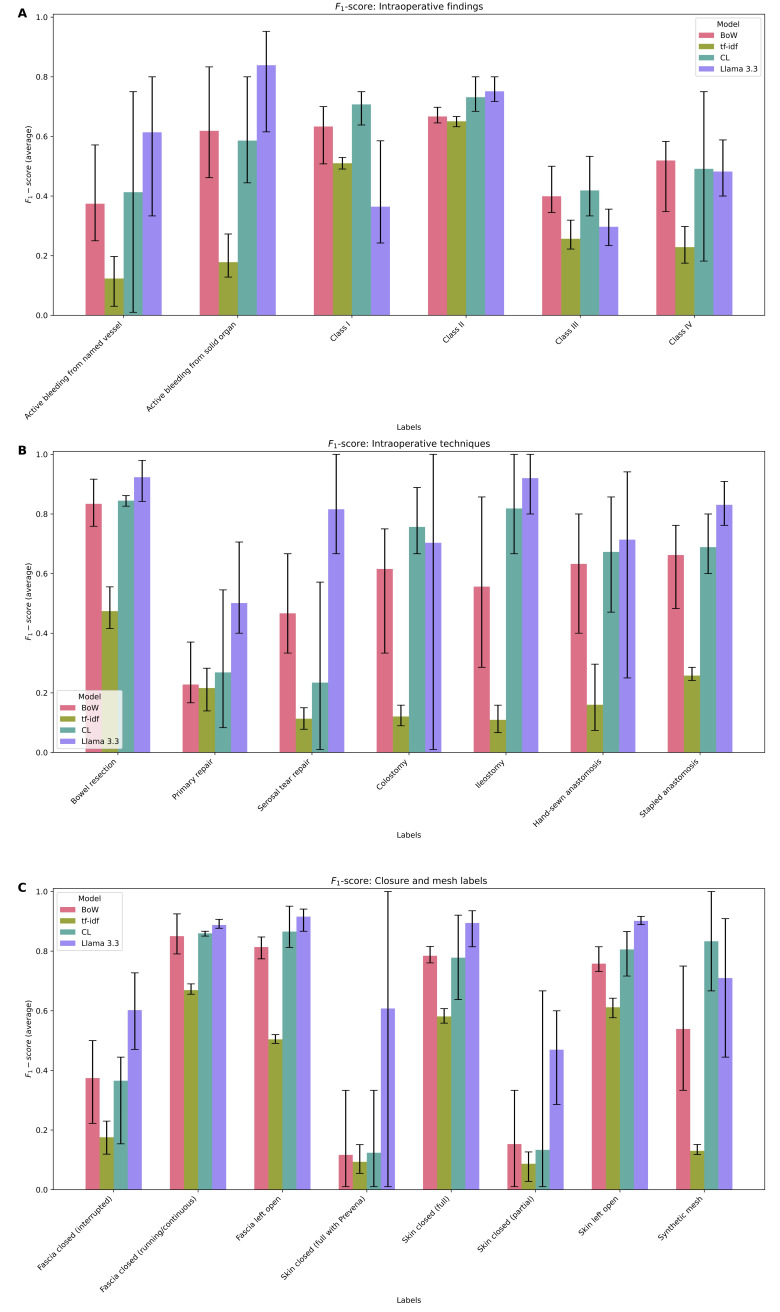
*F*_1_-scores with error bars representing range over 5-fold cross-validation. BoW: bag-of-words; CL: Clinical-Longformer; ti-idf: term frequency-inverse document frequency.

*F*_1_-scores with ranges for the individual labels are visualized in Figure 2 and shown numerically in Tables 3-5. Intraoperative bleeding was well categorized by the Llama model, while surgical wound class was often better served by Clinical-Longformer or BoW models (Figure 2A). For the intraoperative technique (Figure 2B), the Llama model was the highest performer, with the Clinical-Longformer and BoW models performing with overlapping *F*_1_-scores. Intraoperative and skin and fascial closure techniques were best served by the generative model. We noted excellent performance for the Llama 3.3 model in several categories with *F*_1_-scores ≥0.8. Of note, there was surprisingly poor performance on the Prevena label across all models, given that the brand name should often cue a positive class. Interrupted fascial closure was also noticeably poor, despite how this is often specifically stated in the operative note.

Tables 3-5 demonstrate numeric values of the *F*_1_-scores alongside sensitivity and PPVs. The Llama model was again the best performing overall with the notable exception of class II and stapled anastomosis labels. While the PPV of Llama was overall better, it performed poorly in 2 skin closure tasks, class III contamination task, and stapled anastomosis task. Full metrics across all models and labels are shown in Table S3 in Multimedia Appendix 3.

**Table 3.** Comparison of model performance across performance metrics for intraoperative findings.

	Intraoperative findings					
	Active bleeding from named vessel	Active bleeding from solid organ	Class I	Class II	Class III	Class IV
*F*_1_-score						
BoW^a^	0.31	0.64	0.62	0.62	0.35	0.42
tf-idf^b^	0.36	0.6	0.66	0.68	0.41	0.48
CL^c^	0.44	0.63	0.73	0.72	0.46	0.48
Llama	0.61	0.84	0.36	0.75	0.3	0.48
SN^d^						
BoW	0.27	0.61	0.71	0.66	0.35	0.43
tf-idf	0.55	0.8	0.89	0.86	0.55	0.59
CL	0.55	0.72	0.77	0.76	0.54	0.53
Llama	0.55	0.96	0.24	0.9	1	0.95
PPV^e^						
BoW	0.37	0.71	0.56	0.59	0.38	0.46
tf-idf	0.42	0.51	0.53	0.56	0.34	0.41
CL	0.51	0.58	0.69	0.68	0.41	0.46
Llama	0.72	0.77	0.79	0.65	0.18	0.33

a BoW: bag-of-words.

b tf-idf: term frequency-inverse document frequency.

c CL: Clinical-Longformer.

d SN: sensitivity.

e PPV: positive predictive value.

**Table 4.** Comparison of model performance across performance metrics for intraoperative techniques.

	Intraoperative techniques						
	Bowel resection	Primary repair	Serosal tear repair	Colostomy	Ileostomy	Hand-sewn anastomosis	Stapled anastomosis
*F*_1_-score							
BoW^a^	0.86	0.22	0.42	0.51	0.45	0.61	0.65
tf-idf^b^	0.81	0.25	0.43	0.65	0.69	0.59	0.61
CL^c^	0.83	0.37	0.37	0.76	0.78	0.65	0.7
Llama	0.92	0.5	0.82	0.7	0.92	0.71	0.83
SN^d^							
BoW	0.86	0.23	0.32	0.42	0.39	0.58	0.66
tf-idf	0.93	0.3	0.39	0.7	0.7	0.72	0.8
CL	0.87	0.42	0.31	0.78	0.79	0.84	0.77
Llama	0.95	0.4	0.95	0.67	0.87	0.73	0.85
PPV^e^							
BoW	0.87	0.22	0.72	0.73	0.63	0.68	0.65
tf-idf	0.72	0.24	0.65	0.65	0.75	0.54	0.5
CL	0.8	0.35	0.6	0.79	0.83	0.56	0.65
Llama	0.9	0.7	0.73	0.75	1	0.71	0.83

a BoW: bag-of-words

b tf-idf: term frequency-inverse document frequency.

c CL: Clinical-Longformer.

d SN: sensitivity.

e PPV: positive predictive value.

**Table 5.** Comparison of model performance across performance metrics for closure and mesh techniques.

	Closure and mesh techniques							
	Fascia closed interrupted	Fascia closed continuous	Fascia left open	Skin closed (Prevena)	Skin closed (full)	Skin closed (partial)	Skin left open	Synthetic mesh
*F*_1_-score								
BoW^a^	0.34	0.86	0.82	0.15	0.78	0.2	0.77	0.55
tf-idf^b^	0.42	0.82	0.78	0.07	0.74	0.12	0.76	0.6
CL^c^	0.31	0.88	0.84	0.13	0.81	0.23	0.8	0.81
Llama	0.6	0.89	0.92	0.61	0.89	0.47	0.9	0.71
SN^d^								
BoW	0.36	0.9	0.88	0.2	0.84	0.19	0.83	0.51
tf-idf	0.55	0.96	0.94	0.1	0.92	0.09	0.91	0.6
CL	0.27	0.95	0.89	0.1	0.91	0.18	0.81	0.87
Llama	0.7	0.83	0.92	0.51	0.88	0.74	0.94	0.87
PPV^e^								
BoW	0.46	0.83	0.77	0.12	0.74	0.23	0.73	0.7
tf-idf	0.4	0.71	0.67	0.05	0.63	0.25	0.66	0.68
CL	0.4	0.83	0.8	0.2	0.73	0.33	0.81	0.8
Llama	0.56	0.95	0.92	0.8	0.91	0.37	0.87	0.62

a BoW: bag-of-words.

b tf-idf: term frequency-inverse document frequency.

c CL: Clinical-Longformer.

d SN: sensitivity.

e PPV: positive predictive value.

### Context

We evaluated performance on the Llama 3.1-70b model with and without the context document. The model performed better overall with the context, with an average improvement of 0.16 in the *F*_1_-score (Figure S2 in Multimedia Appendix 6). The context offered the greatest improvement in serosal tear repair (+0.4) and the context hurt model performance in class III (−0.19) and stapled-anastomosis (−0.08) labels.

### Error Analysis

A manual review of 5 false negative and positive per label in the encoder-only and decoder-only models revealed several trends in errors, though often it was unclear why a model made a particular prediction. Overall, 88 annotations (0.01% of all annotations) were changed upon review, mostly in bowel resection (n=21), hand-sewn anastomosis (n=19), active bleed from solid organ (n=17), and serosal tear repair (n=13).

Examining the 3 overarching categories, for the encoder-only LLM, bleeds were often picked up, though generally any presence of bleeding was marked positive, regardless of its origins. For intraoperative techniques, false negative instances of bowel resection labels had a clear bowel resection performed in the case. False positives, however, occurred when previous bowel resections were mentioned in the operative report. This was especially true for take-back surgeries when the abdomen is left open because of the need for further surgery. For the ostomy concepts, the most common error was secondary to an ileostomy or colostomy take down (as opposed to creation) or a situation in which the bowel was left in discontinuity with discussion in the operative report of placing an ostomy later. For anastomosis, errors were often likely due to the presence of a stapled resection or the use of the stapler to create a common channel. For closure, fascial closure errors occurred in several cases where a thoracotomy was performed in the same operation as a laparotomy, resulting in the closure of one anatomic fascia and not another. Skin closure failures appeared to be confounded when multiple services operated on the same patient. Partial skin closures were underrepresented in the dataset and the model tended to predict partial closure on both full-closure and open skin with equal affinity.

For the decoder-only model, we had Llama provide explanations for its choice and the explanations along with the findings drove changes in prompting strategies. Performance on bleeding was overall excellent, however, “oozing” from an organ bed or resection was often assigned as “active bleed,” which our annotators and prompts were instructed to mark as negative. For intraoperative techniques, there were commonalities in errors with the Clinical-Longformer model, with prior bowel resections, ostomy takedowns, and instances where both stapled and hand-sewn anastomoses were performed in the same operation. Fascial closures were obscured by the presence of interrupted retention sutures. Several open skin closures were marked as both open and partial skin closures. For skin closure with Prevena and with the exception of some runs of Llama 3.3, the model appeared to simply not understand the instructions despite multiple prompting attempts.

Contamination was difficult to assess for both annotators and models and this information is not always clearly stated in operative reports. Identifying breaches in sterile techniques, purulent versus nonpurulent inflammation, and whether entry into a hollow organ resulted in spillage requires careful description. The generative model often assumed any entry to the abdominal cavity made for Class II or above, despite modifications to prompting techniques. Future studies will extract the attending surgeon attestation for ground truth labels of wound class, which may improve model performance.

## Discussion

### Principal Results

Generative LLMs outperformed fine-tuned encoder-only LLMs and traditional NLP models in a multilabel classification task across the majority of labels. Overall *F*_1_-scores ranged from 0.57 for tf-idf to 0.88 for Llama 3.3. On individual labels, we had *F*_1_-scores of ≥0.8 for multiple classes.

Retrospective analyses drive decision support, quality improvement initiatives, and billing workflows, yet they are limited not only by the intensive manual review process but also by the variable interrater reliability with human labeling [35,36]. To overcome these limitations, we frame operative concept identification as a multilabel document classification task and observe that the autoregressive Llama 3.3 model outperformed both traditional NLP techniques, the Longformer encoder model, and previous versions of the Llama herd.

State-of-the-art clinical NLP tasks rely on transformer-based, foundational LLMs [25,37,38]. They have been used in the well-studied NLP tasks of medical questioning and answering [25,39-41], summarization [16,23,42], named-entity recognition [30,43-46], and document classification [23,47-49]. Studies have largely focused on progress notes, histories and physicals, and discharge summaries, with an interest in the concepts of medications, diseases, and social determinants of health. There are fewer studies on operative notes and available research focuses on word embeddings for prediction tasks rather than individual entities [50-52]. Furthermore, even fewer works have been published using state-of-the-art transformers and foundational LLMs in surgery [53]. This is to our detriment as surgeons, as LLMs are capable of zero-shot learning (the ability to perform tasks without prior examples) and, if performing reliably, may obviate the need for manual chart review in retrospective research [38]. To our knowledge, this paper is the first to explore operative concepts using LLMs as a multilabel classification task in surgery.

### Comparison With Prior Work

Compared with other document classification tasks, our model compared well. A previous multilabel documentation task on chest x-ray reports showed that pretrained models had *F*_1_-score ranges of 0.29 to 0.48 [23]. Notably, traditional BoW-based approaches performed well across many classes. This is not surprising, as depending on the concept, the presence of a word or phrase in operative notes is often sufficient to identify it in text. tf-idf likely underperformed compared to BoW due to the dataset size: limited term frequencies and few documents may favor equal representations of words compared with weighted representations [54]. For many tasks, context may simply not be important. For example, negation is less commonly used, as surgeons will typically describe what they did rather than explain what they did not. In terms of the *F*_1_-score, the generative model offered the most benefit in identifying active bleeding, bowel resection, serosal tear repair, and closure techniques, which are highly context-dependent and rely on the integration of up to several sentences of information. Notably, Clinical-Longformer did not offer much benefit over the BoW model. This may be secondary to the fact that Medical Information Mart for Intensive Care-III does not contain comprehensive operative notes [15].

### Limitations

This study has several limitations. First, exploratory laparotomies represent a difficult case for both human and machine understanding. These operations are, by definition, exploratory, often performed in an emergent setting, can require input from multiple surgical services, and present challenging traumatic and aberrant anatomy. Thus, the language may be less consistent than elective procedures. Nevertheless, we chose basic operative concepts and a common procedure to start our investigation into multilabel document classification. Second, understanding operative reports requires highly technical knowledge. Training annotators, including those with clinical experience, presents challenges, and, despite regular review, there may be instances of inaccurate labeling. To maximize the number of notes, we did not perform a second round of interrater reliability testing, though each note was reviewed by the lead author. As with many other studies, this points to the potential for variability in human annotation, and granting consistency of model outputs may show the potential advantages of LLM augmentation for this task. Third, we acknowledge that the 5-fold training and testing mechanism may result in overly optimistic performance in the BoW, tf-idf models, and Clinical-Longformer models. However, despite this, the untrained Llama model still outperformed the three other classifiers. Fourth, during prompt tuning, we evaluated a random limited subset of the data during exploration, raising the possibility of data leakage. However, we did not examine performance metrics during prompt tuning and focused on model reasoning rather than the label choice itself. Fifth, the distribution of predictions varied by label in BoW, tf-idf, and Clinical-Longformer, though many were left-skewed, suggesting low confidence. More data may improve the performance of these models.

### Conclusions

Given the performance of the off-the-shelf generative model, future studies will incorporate multiple labeled datasets from previous and ongoing retrospective studies at our institution with the goal of human-in-the-loop, streamlined extraction of operative concepts integrated into the research workflow. Future work in agentic retrieval augmented generation with hybrid approaches of keyword search and semantic matching may fit this purpose well [55,56]. We noted improvements in model performance using larger Llama models, a trend we expect to continue as more advanced models are released.

While the use of multilabel document classification may be used to reliably capture select operative concepts with LLMs, further investigation of edge cases and alternative model architectures, such as retrieval augmented generation, will be required prior to deployment for research and quality improvement purposes.

## Supplementary material

10.2196/71176 Multimedia Appendix 1 Operative note annotation guideline.

10.2196/71176 Multimedia Appendix 2 Sample operative note annotation.

10.2196/71176 Multimedia Appendix 3 Supplemental tables.

10.2196/71176 Multimedia Appendix 4 Task prompts.

10.2196/71176 Multimedia Appendix 5 Context.

10.2196/71176 Multimedia Appendix 6 Supplemental figures.

10.2196/71176 Checklist 1 TRIPOD+LLM checklist.

## Abbreviations

BERT: Bidirectional Encoder Representations from Transformers
BoW: bag-of-words
HL: hamming loss
LLM: large language model
NLP: natural language processing
PPV: positive predictive value
tf-idf: term frequency-inverse document frequency
TRIPOD+LLM: Transparent Reporting of a Multivariable Prediction Model for Individual Prognosis or Diagnosis+Large Language Model
