# Supplementary material for: Language Models for Multilabel Document Classification of Surgical Concepts in Exploratory Laparotomy Operative Notes: Algorithm Development Study
Source: JMIR Med Inform. 2025 Jul 9;13:e71176. doi: 10.2196/71176 (PMC12266303; doi:10.2196/71176)
Supplement: Multimedia Appendix 1 [file medinform-v13-e71176-s001.docx]

Note Structure (highlight whole section)

- Patient/Staff/Anesthesia Info
  - Highlight sections that include PATIENT NAME, DATE OF SERVICE, ANESTHESIA, etc.
  - This can be found both at the beginning and in the last sentence of a note
- Procedure Performed
  - Short list of procedures, typically numbered
  - Highlight whole section, including section header
  - If present, highlight whole individual procedures for class contamination if they report entering respiratory, alimentary, genital, or urinary tracts
  - If present, highlight whole individual procedures (bowel resection, colostomy, ileostomy, hand-sewn or stapled bowel anastomosis)
  - Highlight mesh if placed
- Preop dx
  - Highlight section containing pre op dx
- Postop dx
  - Highlight section containing post op dx
- Findings:
  - Documents significant observations
  - Highlight whole section, including section header
- Indications for Procedure
  - Documents why the patient is having surgery
  - Highlight whole section, including section header
  - Use this section for whole note annotation at the bottom (was this the first of a series of operations? take back operation? Was it definitive?)
    - Note: if an abdomen is left open, it is almost always an initial or takeback operation
- Description of Procedure/Procedure in Detail/Operation/Operative Description/etc.:
  - Provides a narrative of the operation
  - Highlight whole section, including section header
  - If present, highlight whole phrase or sentence that captures contamination
  - If present, highlight whole phrase or sentence that captures the procedure
  - If present, highlight the whole phrase that includes mesh
  - If present, highlight whole phrase or sentence that includes facial closure
  - If present, highlight whole phrase or sentence that include skin closure
- Disposition
  - Section that states where patient went after OR
- Complications
  - General “None”, but highlight as a separate section
- Unknown/Unsure
  - Highlight if there is a clear section break, but does not fit the other categories
- Ins and Outs; Patient/Staff Info: ignore, these are placeholders

Annotations

- Case Contamination
  - Class 1 wounds are clean. They are uninfected, no inflammation is present, and do not enter respiratory, alimentary, genital, or urinary tracts. **No organ was entered, no pus**
    - **Note**: the case will be assumed clean if there are no annotations for contamination
  - Class 2 wounds are clean-contaminated. These wounds lack unusual contamination. Class 2 wounds enter the respiratory, alimentary, genital, or urinary tracts in a controlled fashion without a major break in sterile technique.
    - Highlight sentences where respiratory, alimentary, genital, or urinary tracts are entered without spillage of enteric contents.
  - Class 3 wounds are contaminated. Surgical wound that is open, fresh (less than 4 hours old) and/or accidental with a major break in sterile technique and/or uncontrolled/gross spillage from the GI track.
    - These include fresh spillage of enteric contents without evidence of longer-term infection (no pus/abscesses). Acute, non-purulent inflammation.
  - Class 4 wounds are dirty-infected. These wounds typically result from improperly cared for traumatic wounds ≥4 hours old. Class 4 wounds demonstrate devitalized tissue, and they most commonly result from microorganisms present in perforated viscera or the operative field.[3]
    - **Needs to have mention of pus, dirt, or long-standing inflammation**
    - Ex-lap for long standing (>4 hours) intra-abdominal contamination with poop or pus or wounds that contain road-side debris
- Bleeding
  - Sentence-level annotation
  - Only highlight if they specifically mention the name of the vessel bleeding
  - Active solid organ bleed (typically liver, spleen, or kidney)
  - Do not highlight for mention of “oozing” or similar terms
- Procedure
  - Highlight sentence or whole phrase
  - Bowel resection
    - This is often mentioned over multiple sentences
      - Stapling of bowel
      - Division of mesentery
      - Stapling of other side of bowel
      - Removal and “passing off the field to pathology”
    - Highlight each sentence individually
  - Colostomy
    - This often reported over multiple sentences
      - Creation of circular incision in the abdominal wall
      - Carrying incision down to fascia
      - Cruciate incision in fascia
      - Bring up bowel through the incision
      - Maturation of the ostomy
    - Determine if colostomy or ileostomy and highlight all sentences accordingly
  - Ileostomy – same for colostomy
    - Use of small bowel
  - Hand-sewn – no mention of stapler
    - Often mentioned over multiple sentences
      - Will generally mention two layered closures
      - Highlight all sentences/phrases
    - Note: “Lembert” sutures may be used in hand-sewn, stapled, or serosal repair. These are used to reinforce the bowel wall. **Do not highlight for anastomosis**
  - Stapled
    - Often mentioned over multiple sentences
      - Will typically mention the GIA stapler (gastro-intestinal anastomosis)
      - Highlight all sentences/phrases
    - Caution: a stapler may be used for bowel resection but not in the anastomosis portion
  - Primary repair
    - Highlight phrase or whole sentence where it mentions primary repair (i.e. they sewed an open whole in the bowel)
    - This is also either a class II or III depending on spillage
  - Serosal injury repair
    - Small tears in the outer bowel wall
    - A case will be clean with presence of only serosal tears as they do not enter the bowel wall
    - Do not highlight for contamination
    - Highlight sentence/phrase for procedure
- Mesh
  - Sometimes used to close the fascia or support it
  - Highlight sentence or phrase
  - Will often mention brand name, recommend search or ask
- Fascia Closure
  - High-light sentence or phrase
  - Running/Continuous
    - “Closed in the usual fashion”
    - PDS or Vicryl
  - Interrupted
    - “PDS figure of eight”/horizontal mattress
  - Fascia left open – AbThera, Bogota bag
    - Do not need to highlight “skin left open” as this is implied
  - Retention sutures
    - These are placed in addition to continuous/interrupted closure to provide extra reinforcement
    - Note will mention “retention” or “additional reinforcement”
    - typically, Vicryl (as opposed to PDS)
- Skin Closure
  - Full
    - Assume unless otherwise specified
    - Can be stapled or sewn (monocryl or “closed in a subcuticular fashion”)
  - Partial – look out for mention of “wicks” or parts “left to heal by secondary intention”
    - “Skin at the umbilicus” – references a partial closure
  - Open – wound vac placement (“black sponge”, “white sponge”, not AbThera), wet-to-dry dressing, heal by secondary intention, “packed”

Surgery Level Annotations:

- Operation Stage
  - Definitive/Final – fascia is closed (unless left open for patient going to palliative)
    - This includes single procedures done after an initial hospital visit (i.e. ostomy takedown or hernia repair following an operation at a previous hospitalization)
  - Initial – first operation, fascia left open, no previous operations mentioned
  - Take back – re-opening recent laparotomy, fascia left open (this can sometimes happen multiple times during a single patient admission)
- Case Contamination
  - Will be highest level highlighted for the case

General Notes

- No sub highlights except for description of procedure
- Don’t highlight negations (“no bowel resection”)
- Don’t highlight intentions (“which was intended for placement of an ostomy”)
- Ok double/triple highlight procedures if multiple concepts present
- Findings are a specific subsection, don’t need to be highlighted in procedure portion
