# Supplementary figures and images for: Language Models for Multilabel Document Classification of Surgical Concepts in Exploratory Laparotomy Operative Notes: Algorithm Development Study
Source: JMIR Med Inform. 2025 Jul 9;13:e71176. doi: 10.2196/71176 (PMC12266303; doi:10.2196/71176)

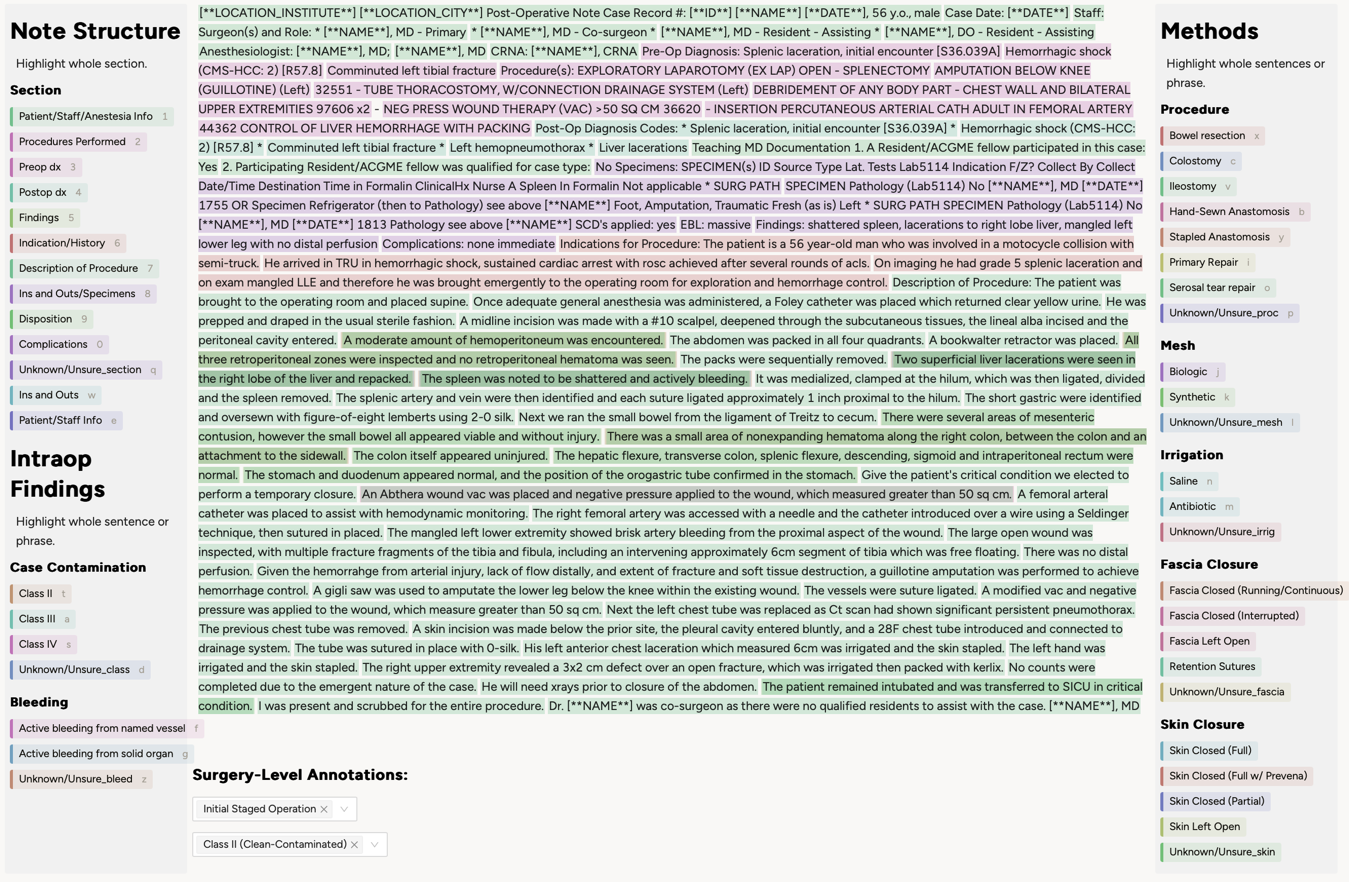

Supplement: Multimedia Appendix 2 [file medinform-v13-e71176-s002.docx]
