# Supplementary material for: Language Models for Multilabel Document Classification of Surgical Concepts in Exploratory Laparotomy Operative Notes: Algorithm Development Study
Source: JMIR Med Inform. 2025 Jul 9;13:e71176. doi: 10.2196/71176 (PMC12266303; doi:10.2196/71176)
Supplement: Multimedia Appendix 3 [file medinform-v13-e71176-s003.docx]

**Table S1. Cohen kappa scores across labels.** Active bleeds, ileostomy, Prevena closure, and interrupted fascial closure were added following the original test.

| Annotator | MS1 | MS2 | MS3 | MS4 | MS5 | MS6 | MS7 |
| --- | --- | --- | --- | --- | --- | --- | --- |
| Bowel resection | 1 | 0.89 | 0.77 | 1 | 1 | 1 | 0.89 |
| Class I | 0.57 | 0.86 | 0.69 | 0.63 | 0.50 | 0.69 | 0.86 |
| Class II | 0.58 | 0.79 | 0.79 | 0.78 | 0.69 | 0.90 | 1 |
| Class III | 0.63 | 0.63 | 0.66 | 0.76 | 0.76 | 0.89 | 0.89 |
| Class IV | 0.64 | 0.64 | 1 | 0.64 | 1 | 1 | 1 |
| Colostomy | 0.46 | 1 | 0 | 0.77 | 0.77 | 0.46 | 0.61 |
| Fascia Closed (Running/Continuous) | 0.79 | 0.89 | 0.89 | 0.89 | 0.78 | 0.90 | 0.79 |
| Fascia Left Open | 0.90 | 0.80 | 0.90 | 0.90 | 0.80 | 1 | 0.90 |
| Hand-Sewn Anastomosis | 0.62 | 0.64 | 0.64 | 0.50 | 0.64 | 1 | 0.77 |
| Primary Repair | 0 | 0.64 | 0 | 0.64 | 0.50 | 0.64 | 0.44 |
| Serosal tear repair | 0.32 | 0.46 | 0.46 | 0.77 | 0.77 | 1 | 0.46 |
| Skin Closed (Full) | 1 | 0.74 | 0.83 | 1 | 0.63 | 1 | 0.74 |
| Skin Closed (Partial) | 1 | 1 | 1 | 1 | 0 | 1 | 0.64 |
| Skin Left Open | 1 | 0.46 | 0.64 | 0.64 | 0.46 | 0.64 | 0.64 |
| Stapled Anastomosis | 0 | 1 | 1 | 1 | 1 | 1 | 0.64 |

**Table S2. Proportion of each label in each fold using iterative stratification.**

|  | Fold 1 | | Fold 2 | | Fold 3 | | Fold 4 | | Fold 5 | |
| --- | --- | --- | --- | --- | --- | --- | --- | --- | --- | --- |
|  | Train | Test | Train | Test | Train | Test | Train | Test | Train | Test |
| Active bleeding from named vessel | 0.05 | 0.08 | 0.05 | 0.08 | 0.06 | 0.03 | 0.05 | 0.06 | 0.06 | 0.02 |
| Active bleeding from solid organ | 0.09 | 0.09 | 0.08 | 0.14 | 0.10 | 0.05 | 0.09 | 0.11 | 0.10 | 0.07 |
| Bowel resection | 0.32 | 0.28 | 0.30 | 0.34 | 0.31 | 0.31 | 0.30 | 0.36 | 0.32 | 0.27 |
| Class I | 0.34 | 0.36 | 0.34 | 0.36 | 0.34 | 0.35 | 0.34 | 0.34 | 0.35 | 0.31 |
| Class II | 0.48 | 0.49 | 0.47 | 0.52 | 0.48 | 0.49 | 0.49 | 0.46 | 0.49 | 0.45 |
| Class III | 0.15 | 0.15 | 0.15 | 0.14 | 0.14 | 0.20 | 0.16 | 0.10 | 0.15 | 0.16 |
| Class IV | 0.14 | 0.09 | 0.13 | 0.12 | 0.13 | 0.12 | 0.11 | 0.19 | 0.13 | 0.12 |
| Colostomy | 0.05 | 0.04 | 0.05 | 0.07 | 0.05 | 0.07 | 0.05 | 0.05 | 0.06 | 0.04 |
| Fascia Closed (Interrupted) | 0.11 | 0.04 | 0.09 | 0.14 | 0.10 | 0.08 | 0.09 | 0.10 | 0.09 | 0.12 |
| Fascia Closed (Running/Continuous) | 0.50 | 0.52 | 0.50 | 0.53 | 0.50 | 0.51 | 0.51 | 0.49 | 0.51 | 0.47 |
| Fascia Left Open | 0.33 | 0.35 | 0.33 | 0.36 | 0.33 | 0.35 | 0.34 | 0.33 | 0.34 | 0.31 |
| Hand-Sewn Anastomosis | 0.08 | 0.12 | 0.08 | 0.10 | 0.09 | 0.08 | 0.09 | 0.06 | 0.09 | 0.07 |
| Ileostomy | 0.04 | 0.08 | 0.05 | 0 | 0.04 | 0.05 | 0.04 | 0.05 | 0.05 | 0.04 |
| Primary Repair | 0.12 | 0.13 | 0.12 | 0.11 | 0.13 | 0.09 | 0.13 | 0.10 | 0.11 | 0.17 |
| Serosal tear repair | 0.06 | 0.07 | 0.06 | 0.04 | 0.06 | 0.05 | 0.06 | 0.06 | 0.06 | 0.07 |
| Skin Closed (Full w/ Prevena) | 0.04 | 0.04 | 0.04 | 0.05 | 0.05 | 0.01 | 0.04 | 0.03 | 0.03 | 0.06 |
| Skin Closed (Full) | 0.41 | 0.41 | 0.41 | 0.42 | 0.41 | 0.43 | 0.41 | 0.41 | 0.42 | 0.37 |
| Skin Closed (Partial) | 0.04 | 0.04 | 0.03 | 0.08 | 0.05 | 0.03 | 0.04 | 0.04 | 0.05 | 0.02 |
| Skin Left Open | 0.44 | 0.43 | 0.43 | 0.49 | 0.43 | 0.47 | 0.45 | 0.41 | 0.45 | 0.41 |
| Stapled Anastomosis | 0.15 | 0.15 | 0.15 | 0.15 | 0.15 | 0.13 | 0.14 | 0.16 | 0.15 | 0.14 |
| Synthetic | 0.05 | 0.09 | 0.05 | 0.08 | 0.05 | 0.07 | 0.07 | 0.03 | 0.07 | 0.02 |

**Table S3. Complete model performance metrics.**

| Label | AUROC | | | | AUPRC | | | | F1 | | | | Sensitivity | | | | PPV | | | |
| --- | --- | --- | --- | --- | --- | --- | --- | --- | --- | --- | --- | --- | --- | --- | --- | --- | --- | --- | --- | --- |
|  | BoW | tfidf | CL | Llama | BoW | tfidf | CL | Llama | BoW | tfidf | CL | Llama | BoW | tfidf | CL | Llama | BoW | tfidf | CL | Llama |
| Active bleeding  from named vessel | 0.89 | 0.91 | 0.89 | 0.77 | 0.46 | 0.44 | 0.46 | 0.44 | 0.31 | 0.36 | 0.44 | 0.61 | 0.27 | 0.55 | 0.55 | 0.55 | 0.37 | 0.42 | 0.51 | 0.72 |
| Active bleeding  from solid organ | 0.96 | 0.96 | 0.93 | 0.97 | 0.67 | 0.72 | 0.75 | 0.74 | 0.64 | 0.6 | 0.63 | 0.84 | 0.61 | 0.8 | 0.72 | 0.96 | 0.71 | 0.51 | 0.58 | 0.77 |
| Class I | 0.79 | 0.81 | 0.86 | 0.61 | 0.67 | 0.68 | 0.79 | 0.46 | 0.62 | 0.66 | 0.73 | 0.36 | 0.71 | 0.89 | 0.77 | 0.24 | 0.56 | 0.53 | 0.69 | 0.79 |
| Class II | 0.68 | 0.7 | 0.77 | 0.72 | 0.66 | 0.69 | 0.73 | 0.63 | 0.62 | 0.68 | 0.72 | 0.75 | 0.66 | 0.86 | 0.76 | 0.90 | 0.59 | 0.56 | 0.68 | 0.65 |
| Class III | 0.69 | 0.76 | 0.82 | 0.59 | 0.38 | 0.43 | 0.47 | 0.18 | 0.35 | 0.41 | 0.46 | 0.30 | 0.35 | 0.55 | 0.54 | 1.00 | 0.38 | 0.34 | 0.41 | 0.18 |
| Class IV | 0.85 | 0.87 | 0.85 | 0.82 | 0.5 | 0.56 | 0.51 | 0.32 | 0.42 | 0.48 | 0.48 | 0.48 | 0.43 | 0.59 | 0.53 | 0.95 | 0.46 | 0.41 | 0.46 | 0.33 |
| Bowel resection | 0.95 | 0.95 | 0.96 | 0.95 | 0.92 | 0.93 | 0.93 | 0.87 | 0.86 | 0.81 | 0.83 | 0.92 | 0.86 | 0.93 | 0.87 | 0.95 | 0.87 | 0.72 | 0.8 | 0.90 |
| Primary Repair | 0.63 | 0.68 | 0.7 | 0.69 | 0.26 | 0.28 | 0.37 | 0.36 | 0.22 | 0.25 | 0.37 | 0.50 | 0.23 | 0.3 | 0.42 | 0.40 | 0.22 | 0.24 | 0.35 | 0.70 |
| Serosal tear repair | 0.85 | 0.88 | 0.86 | 0.96 | 0.48 | 0.54 | 0.52 | 0.70 | 0.42 | 0.43 | 0.37 | 0.82 | 0.32 | 0.39 | 0.31 | 0.95 | 0.72 | 0.65 | 0.6 | 0.73 |
| Colostomy | 0.97 | 0.97 | 0.99 | 0.83 | 0.69 | 0.73 | 0.87 | 0.64 | 0.51 | 0.65 | 0.76 | 0.70 | 0.42 | 0.7 | 0.78 | 0.67 | 0.73 | 0.65 | 0.79 | 0.75 |
| Ileostomy | 0.95 | 0.94 | 0.88 | 0.93 | 0.62 | 0.74 | 0.85 | 0.87 | 0.45 | 0.69 | 0.78 | 0.92 | 0.39 | 0.7 | 0.79 | 0.87 | 0.63 | 0.75 | 0.83 | 1.00 |
| Hand-Sewn Anastomosis | 0.91 | 0.94 | 0.96 | 0.85 | 0.67 | 0.61 | 0.7 | 0.59 | 0.61 | 0.59 | 0.65 | 0.71 | 0.58 | 0.72 | 0.84 | 0.73 | 0.68 | 0.54 | 0.56 | 0.71 |
| Stapled Anastomosis | 0.91 | 0.92 | 0.93 | 0.91 | 0.75 | 0.75 | 0.82 | 0.72 | 0.65 | 0.61 | 0.7 | 0.83 | 0.66 | 0.8 | 0.77 | 0.85 | 0.65 | 0.5 | 0.65 | 0.83 |
| Fascia Closed (Interrupted) | 0.78 | 0.82 | 0.77 | 0.82 | 0.45 | 0.43 | 0.4 | 0.42 | 0.34 | 0.42 | 0.31 | 0.60 | 0.36 | 0.55 | 0.27 | 0.70 | 0.46 | 0.4 | 0.4 | 0.56 |
| Fascia Closed (Running/Continuous) | 0.89 | 0.89 | 0.92 | 0.89 | 0.86 | 0.86 | 0.9 | 0.88 | 0.86 | 0.82 | 0.88 | 0.89 | 0.9 | 0.96 | 0.95 | 0.83 | 0.83 | 0.71 | 0.83 | 0.95 |
| Fascia Left Open | 0.93 | 0.93 | 0.93 | 0.93 | 0.86 | 0.88 | 0.88 | 0.88 | 0.82 | 0.78 | 0.84 | 0.92 | 0.88 | 0.94 | 0.89 | 0.92 | 0.77 | 0.67 | 0.8 | 0.92 |
| Skin Closed  (Full w/ Prevena) | 0.82 | 0.86 | 0.81 | 0.76 | 0.22 | 0.26 | 0.25 | 0.53 | 0.15 | 0.07 | 0.13 | 0.61 | 0.2 | 0.1 | 0.1 | 0.51 | 0.12 | 0.05 | 0.2 | 0.80 |
| Skin Closed (Full) | 0.89 | 0.89 | 0.9 | 0.91 | 0.84 | 0.85 | 0.85 | 0.85 | 0.78 | 0.74 | 0.81 | 0.89 | 0.84 | 0.92 | 0.91 | 0.88 | 0.74 | 0.63 | 0.73 | 0.91 |
| Skin Closed (Partial) | 0.6 | 0.69 | 0.74 | 0.84 | 0.25 | 0.26 | 0.25 | 0.27 | 0.2 | 0.12 | 0.23 | 0.47 | 0.19 | 0.09 | 0.18 | 0.74 | 0.23 | 0.25 | 0.33 | 0.37 |
| Skin Left Open | 0.89 | 0.89 | 0.9 | 0.91 | 0.86 | 0.87 | 0.88 | 0.84 | 0.77 | 0.76 | 0.8 | 0.90 | 0.83 | 0.91 | 0.81 | 0.94 | 0.73 | 0.66 | 0.81 | 0.87 |
| Synthetic | 0.94 | 0.97 | 0.97 | 0.92 | 0.66 | 0.8 | 0.84 | 0.57 | 0.55 | 0.6 | 0.81 | 0.71 | 0.51 | 0.6 | 0.87 | 0.87 | 0.7 | 0.68 | 0.8 | 0.62 |
