# Supplementary material for: Language Models for Multilabel Document Classification of Surgical Concepts in Exploratory Laparotomy Operative Notes: Algorithm Development Study
Source: JMIR Med Inform. 2025 Jul 9;13:e71176. doi: 10.2196/71176 (PMC12266303; doi:10.2196/71176)
Supplement: Multimedia Appendix 4 [file medinform-v13-e71176-s004.docx]

Task: Extract the presence of an active bleed from a named vessel above operative note.

Instructions:

- Focus on the section where the surgical findings and details are described.

- Look for mentions of active bleeding from large arteries or veins with specific names (e.g., iliac, vena cava, aorta, iliac, renal, superior mesenteric artery, short gastric etc.).

- First look for mention of a named vessel, then look for presence of active bleeding

- Must mention both the name and have active bleeding

- Must not mention only a named vessel or only an active bleed

- Do not include any bleeding from solid organs (e.g., kidney, spleen, liver)

- Identify if such bleeding is mentioned.

- Report findings as either 1 (yes) or 0 (no)

- Explain the rationale behind your answer

- Here are a few examples:

'We noted bleeding from a short gastric which was oversewn with a single figure-of-eight 2.0 silk suture.'

'We had some bleeding in this area related to a branch coming off the portal vein'

'It was decided that the patient would need to be placed emergently on bypass in order to repair this large rent in the superior vena cava'

Task: Extract the presence of an active bleed from a solid organ in the above operative note.

Instructions:

- Focus on the section where the surgical findings and details are described.

- Look for mentions of active bleeding from solid organs (e.g., liver, spleen, kidney, adrenal glands, pancreas, ovaries, uterus).

- Look for mentions of capsular tears, lacerations, or damage of solid organs

- Ignore injuries to hollow organs (e.g., stomach, bowel, bladder, ureters)

- Identify if such bleeding is mentioned.

- Report findings as either 1 (yes) or 0 (no)

- Explain the rationale behind your answer

- Here are a few examples:

'Mild bleeding from the liver was noted'

'Large injury to the kidney'

'An obliterated right lobe of the liver corresponding to the gunshot'

Task: Determine if a bowel resection was performed in the above operative note:

Instructions:

- Focus on the section where the surgical findings and details are described.

- Look for mentions of bowel resection (e.g., esophagus, stomach, large intestine, small

intestine, colon)

- DO NOT include resection performed at a previous operation

- Look for explicit mentions of bowel resection, do not infer

- Hint: if a bowel resection is performed this operation, the surgeon will describe the use of a stapling device to perform the resection

- Hint: often, an anastomosis is performed at a second operation after a different surgery for a bowel resection, do not label "bowel resection" just because there was an anastomosis

- Hint: an ostomy does not always involve a bowel resection

- Hint: a primary repair of a hole in the bowel is not a bowel resection

- Identify if such a procedure was mentioned.

- Report findings as either 1 (yes) or 0 (no)

- Explain the rationale behind your answer

Task: Determine if a colostomy was placed in the above operative note (excluding ileostomy).

Instructions:

- Focus on the section where the surgical findings and details are described.

- Look for mentions of colostomy placement using the large bowel (ascending, transverse, descending, sigmoid)

- Do not include ileostomies

- Hint: there will be a specific description of maturing the ostomy, typically making a quarter-sized hole with brooking stitches

- Identify if such a procedure was mentioned.

- Report findings as either 1 (yes) or 0 (no)

Task: Determine if an ileostomy was placed in the above operative note (excluding colostomy).

Instructions:

- Focus on the section where the surgical findings and details are described.

- This one is a difficult Task, be very careful and question your response

- DO NOT include ileostomies placed at prior operations

- DO NOT include ileostomy take downs, where the ileostomy is removed, and an anastomosis is performed to restore normal anatomy

- The surgeon will generally describe maturing an ileostomy with a separate incision and sutures

- Look for mentions of ileostomy placement using the small bowel (exclude mentions of colostomy).

- Ileal conduits are not ileostomies

- Identify if such a procedure was mentioned.

- Report findings as either 1 (yes) or 0 (no)

Task: Determine if the abdominal wall fascia was closed in an interrupted fashion in the above operative note (excluding cases where it was left open, closed with an AbThera, or closed in a continuous fashion):

Instructions:

- Focus on the section where the surgical findings and details are described.

- Look for mentions of the fascia (sometimes called midline) being closed in an interrupted fashion

- If fascia is closed, it is either closed in an interrupted or running/continuous fashion, not both.

- Report findings as either 1 (yes) or 0 (no)

- Explain the rationale behind your answer

Task: Determine if the abdominal wall fascia was closed in a continuous fashion in the above operative note (excluding cases where it was closed in an interrupted fashion, left open, or closed with an AbThera):

Instructions:

- Focus on the section where the surgical findings and details are described.

- If fascia is closed, it is either closed in an interrupted or running/continuous fashion, not both

- Look for mentions of the fascia (sometimes called midline) being closed in a running/continuous fashion

- Exclude mentions of fascia being left open, closed with an AbThera, or closed in an interrupted fashion.

- If it is not clear whether an interrupted or running/continuous closure was performed, assign it to a running/continuous fashion as this is most common

- Report findings as either 1 (yes) or 0 (no)

- Explain the rationale behind your answer

Task: Determine if the abdominal wall fascia was left open in the above operative note (including cases with AbThera placement and no skin closure).

Instructions:

- Focus on the section where the surgical findings and details are described.

- Look for absence of mention of fascial closure with interrupted or continuous suture

- Look for mentions of the fascia (sometimes called midline) being left open or AbThera placement or temporary abdominal dressing

- Exclude mentions of fascia being closed in a continuous or interrupted fashion.

- Identify if such a condition was mentioned.

- Report findings as either 1 (yes) or 0 (no)

- Explain the rationale behind your answer

Task: Determine if a bowel anastomosis was performed in the above operative note and if it was performed using a hand-sewn technique with just sutures (not stapled). Note that any mention of a hand-sewn anastomosis should take precedence over a stapled anastomosis.

Instructions:

- Focus on the section where the surgical findings and details are described.

- Look for mentions of a bowel anastomosis being performed. Do not include choledochostomies, ileostomies, or colostomies.

- Refer to the context to see different descriptions between hand-sewn and stapled-anastomosis

- Each anastomosis will either be hand-sewn or stapled

- There can be multiple hand-sewn and/or stapled anastomoses performed in the same operation. Both should be recorded as a positive class if both are present.

- The author will either directly state "hand-sewn" or will imply it closing the bowel in two layers or running a suture, either as side-to-side or end-to-end or end-to-side.

- Sometimes, stapled anastomoses are reinforced with sutures, this is NOT a hand-sewn anastomosis

- Report findings as either 1 (yes) or 0 (no)

- Explain the rationale behind your answer

Task: Determine if a bowel anastomosis was performed in the above operative note and if it was performed using a stapled technique (not hand-sewn).

Instructions:

- Focus on the section where the surgical findings and details are described.

- Look for mentions of a bowel anastomosis being performed. Do not include choledochostomies, ileostomies, or colostomies.

- Refer to the prompt to see different descriptions between hand-sewn and stapled-anastomosis

- Each anastomosis will either be stapled or hand-sewn, not both

- There can be multiple hand-sewn and/or stapled anastomoses performed in the same operation. Both should be recorded as a positive class if both are present.

- The author will generally state "a stapled anastomosis was performed" either as side-to-side or end-to-end or end-to-side.

- Sometimes, stapled anastomoses are reinforced with sutures, this is a stapled anastomosis

- Report findings as either 1 (yes) or 0 (no)

- Explain the rationale behind your answer

Task: Extract the presence of a serosal tear repair in the above operative note.

Instructions:

- Focus on the section where the surgical findings and details are described.

- Look for mentions of serosal tear repair. This is where an injury to the bowel occurred, but it was only partial thickness.

- Examples will mention damage to the serosa. Typically repair with Lembert sutures or imbricating stitch.

- This is different from a primary repair, where there is a full thickness hole in the bowel

- Identify if such a repair is mentioned.

- Report findings as either 1 (yes) or 0 (no)

- Explain the rationale behind your answer

Task: Extract the presence of a primary bowel repair in the above operative note:

Instructions:

- Focus on the section where the surgical findings and details are described.

- Look for mentions of primary bowel repair. This is different from serosal tear. Here there is full-thickness injury to the bowel where the intestine is entered, either surgically or through a penetrating wound.

- Identify if such a repair is mentioned. This will mention repair with suture.

- Remember: an anastomosis is not performed during primary repair but may be performed elsewhere in the operation.

- Report findings as either 1 (yes) or 0 (no)

- Explain the rationale behind your answer

Task: Extract full skin closure in the following operative note:

Instructions:

- Focus on the section where the surgical findings and details are described.

- Look for mentions of skin closure and identify if it was fully closed as opposed to being left open or partially closed.

- full closure will be performed with monocryl or staples.

- You will find this information at the end of the operative note.

- Report findings as either 1 (yes) or 0 (no)

- Explain the rationale behind your answer

Task: Extract partial skin closure in the following operative note:

Instructions:

- Focus on the section where the surgical findings and details are described.

- Look for mentions of skin closure and identify if it was partially closed as opposed to being fully open or fully closed.

- Partially closed will be explicitly stated using the word "partial" or a synonym

- It can be performed with large gaps between staples or with only the inferior or superior aspect of the wound closed

- You will find this information at the end of the operative note.

- Report findings as either 1 (yes) or 0 (no)

- Explain the rationale behind your answer

Task: Extract open skin closure in the following operative note:

Instructions:

- Focus on the section where the surgical findings and details are described.

- Look for mentions of skin closure and identify if it was left open as opposed to being partially closed or fully closed.

- left open will either be stated explicitly or implicitly with use of a packed dressing (wet to dry) or wound vac. If the fascia is left open, so is the skin.

- You will find this information at the end of the operative note.

- Report findings as either 1 (yes) or 0 (no)

- Explain the rationale behind your answer

Task: Extract skin closure with Prevena in the following operative note:

Instructions:

- Focus on the section where the surgical findings and details are described.

- Look for mentions of skin closure. If the skin is fully closed identify if a Prevena was also placed.

- Prevena is type of wound vac, it superficial and goes over only fully closed skin. This is different than an incisional vac that goes in open wounds, or AbThera that go in open abdomens with open fascia.

- You will find this information at the end of the operative note.

- Report findings as either 1 (yes) or 0 (no)

- Explain the rationale behind your answer

Task: Extract the presence of a synthetic mesh used in the above operative note:

Instructions:

- Focus on the section where the surgical findings and details are described.

- Look for mentions of mesh use and identify if it was synthetic mesh.

- synthetic mesh is polypropylene, ePTFE, Vicryl, or polester

- biologic meshes include alloderm, strattice, surgisis

- ONLY report 1 if a synthetic mesh is mentioned

- Report findings as either 1 (presence of synthetic mesh) or 0 (absence)

- Explain the rationale behind your answer

Task: Extract if there is a Class 1 wound from the operative note.

- Focus specifically on identifying clean (Class 1) wounds based on the wound classification system.

- Entry into the abdominal or thoracic cavity, by itself, does NOT result in a classification above Class 1.

- Class 1 applies even in laparotomy or thoracotomy cases, if there is no entry into the respiratory, alimentary, genital, or urinary tracts, and no spillage or contamination.

- Do NOT classify as Class 2 unless there is clear evidence of entry into or perforation of the respiratory, alimentary, genital, or urinary tracts under controlled conditions, without spillage.

- Provide clear justification for why the wound is considered Class 1 or not.

Task: Extract if there is a Class 2 wound from the operative note

- Refer to the wound classification system described in the prompt

- Report findings as either 1 (Class 2 wound present) or 0 (absent)

- Explain the rationale behind your answer

Task: Extract if there is a Class 3 wound from the operative note

- Refer to the wound classification system described in the prompt

- Report findings as either 1 (Class 3 wound present) or 0 (absent)

- Explain the rationale behind your answer

Task: Extract if there is a Class 4 wound from the operative note

- Refer to the wound classification system described in the prompt

- Report findings as either 1 (Class 4 wound present) or 0 (absent)

- Explain the rationale behind your answer
