# Supplementary material for: Language Models for Multilabel Document Classification of Surgical Concepts in Exploratory Laparotomy Operative Notes: Algorithm Development Study
Source: JMIR Med Inform. 2025 Jul 9;13:e71176. doi: 10.2196/71176 (PMC12266303; doi:10.2196/71176)
Supplement: Multimedia Appendix 5 [file medinform-v13-e71176-s005.docx]

There are two types of mesh: synthetic and biologic. Synthetic companies include Ethicon company (Prolene hernia system, Ultrapro mesh, Physiomesh, Vicryl mesh) and Gore company (dualmesh, synecor, bio-a). Biologic mesh includes LifeCell company (strattice and alloderm) and Stryker (DermACELL).

There are three types of skin closure: full skin closure, partial skin closure, and skin not closed. Full skin closure is performed either by staples or using suture material with no gaps left in the skin. Partial skin closure is indicated by closing only half of the incision or using staples and sutures intermittently to have large gaps in between which are often packed with kerlix or iodine-soaked gauze strips. Skin is left open when closure is not mentioned or when the entire wound is packed with a wound vac or a soaked-kerlix with a dry dressing. Open skin is also present when the abdomen is temporarily closed with an AbThera, and the fascia is not sutured together.

Primary repair of the bowel happens when no bowel is resected, and the edges of the full-thickness defect are brought together with suture. In serosal tear repair, the defect is only in the serosal layer, and this is also repaired with a suture without bowel resection.

Colostomy and ileostomy are different procedures and need to record separately. Colostomy is formed from the ascending, transverse, descending, or sigmoid colon. Ileostomy is formed from the small bowel.

Bowel anastomosis can is considered either hand-sewn or stapled, though stapled anastomosis will often include hand-sewn components, and the bowel may be respected, but not anastomosed, with staplers. Use of a stapler within the lumen of the bowel constitutes a stapled anastomosis. Be sure to identify whether the stapler (either linear or circular) was used in the anastomosis part and not the resection part. Here is a description of a stapled technique:

For a stapled anastomosis, two small enterotomies are created in the antimesenteric borders of the intestine. One fork of a linear stapler is placed in each intestinal lumen and then fired. The common enterotomy is then closed with either a TA stapling device or a full thickness running monofilament suture after inspection of the staple line for hemostasis.

For a two-layer hand-sewn anastomosis, the posterior layer is created by suturing the antimesenteric side of the small bowel to the inferior staple line of the pouch. Enterotomies are made using scissors with electrocautery on the anterior wall of the pouch and the antimesenteric side of the intestine. An inner layer can be performed with two sutures beginning in the middle of the enterotomy consisting of full-thickness bites of the posterior row in each direction ensuring inclusion of both corners. An outer layer of Lembert sutures is placed in interrupted or running fashion to complete the anastomosis.

Active bleeding from named vessels includes specific mention of blood vessel names, including but not limited to superior mesenteric artery (SMA), inferior mesenteric artery (IMA), short gastric arteries, splenic artery, renal artery, superior mesenteric vein (SMV), gastroduodenal artery, inferior mesenteric vein (SMV), cystic artery, and iliac artery and vein. This also includes branches of named vessels. Active bleeding from a named vessel is only present if the note contains the name of the vessel with a description of bleeding and not if there is only bleeding with no named vessel or just a description of the blood vessel with no bleeding.

Surgical wound classes are defined as follows:

- Class 1 wounds are categorized as clean wounds. These types of wounds are not infected, do not exhibit any signs of inflammation, and are typically closed.

If drainage is required, a closed draining approach is recommended.

It is worth noting that Class 1 wounds do not involve entry into the respiratory,

alimentary, genital, or urinary tracts. Examples of clean wounds include

an inguinal hernia repair, a thyroidectomy, laparotomy without enterotomy or bowel resection, and thoracotomy without resection of lung or esophagus

- Class 2 wounds are categorized as clean-contaminated, which means they have a low level of contamination. These types of wounds involve entry into the respiratory, alimentary, genital, or urinary tracts but only under controlled circumstances without spillage of enteric contents.

- Class 3 wounds are classified as contaminated and typically result from a breach in sterile techniques or leakage from the gastrointestinal tract. Incisions resulting from acute or non-purulent inflammation are also considered Class 3 wounds.

- Class 4 wounds are dirty or infected. These injuries usually occur from

inadequate treatment of traumatic wounds, gross purulence, and evident infections. When tissues lose vitality, it can lead to Class 4 wounds. This is often caused by surgery or microorganisms found in perforated organs
