# Supplementary material for: Language Models for Multilabel Document Classification of Surgical Concepts in Exploratory Laparotomy Operative Notes: Algorithm Development Study
Source: JMIR Med Inform. 2025 Jul 9;13:e71176. doi: 10.2196/71176 (PMC12266303; doi:10.2196/71176)

**Figure S1. Comparison of Llama model performance based on size for multi-label classification across the intraoperative findings (top panel), intraoperative techniques (middle panel), and closure techniques (bottom panel).**


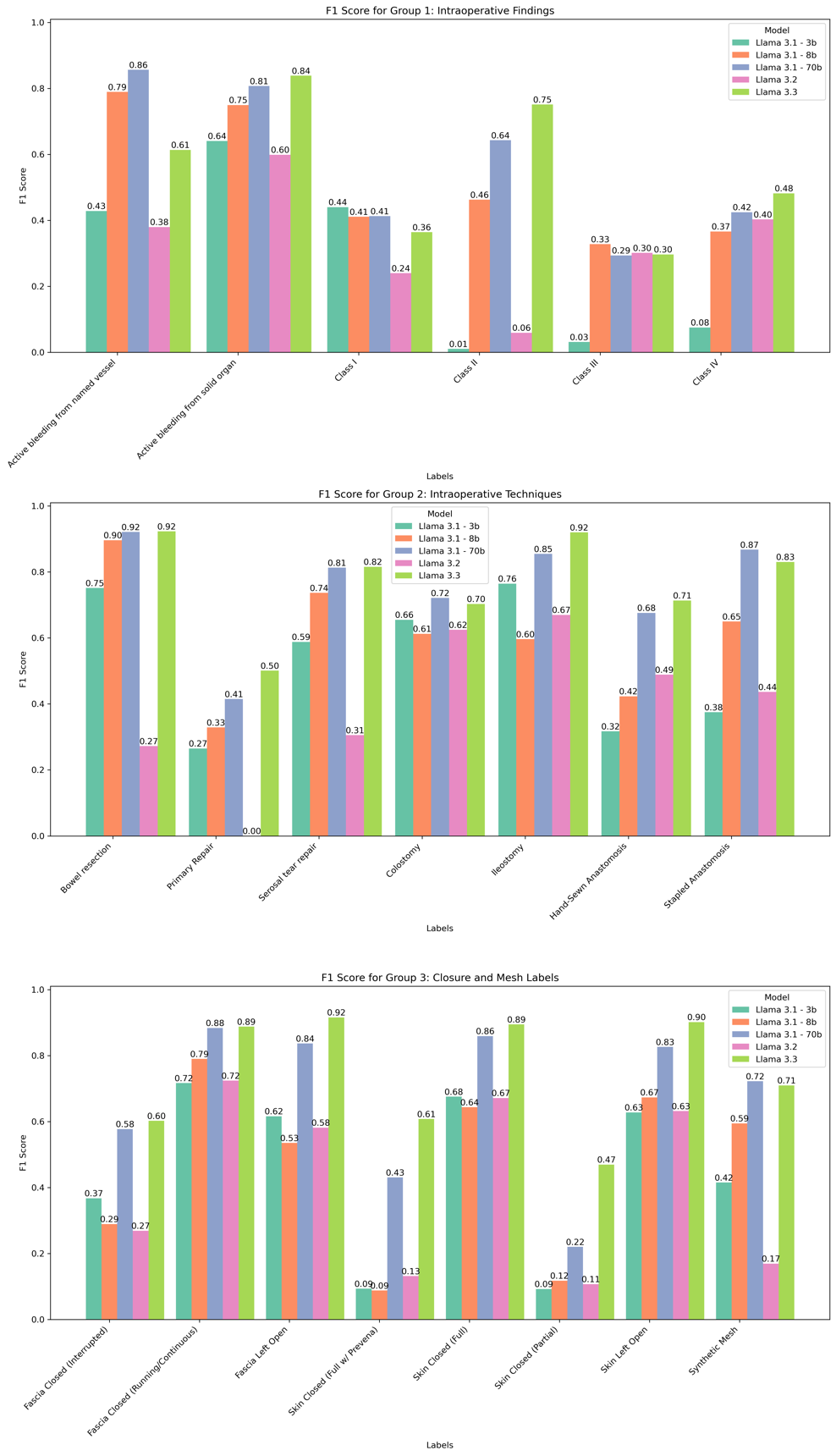


**Figure S2. Comparison of model performance using F1 scores using Llama 3.1 70B model with and without context.**


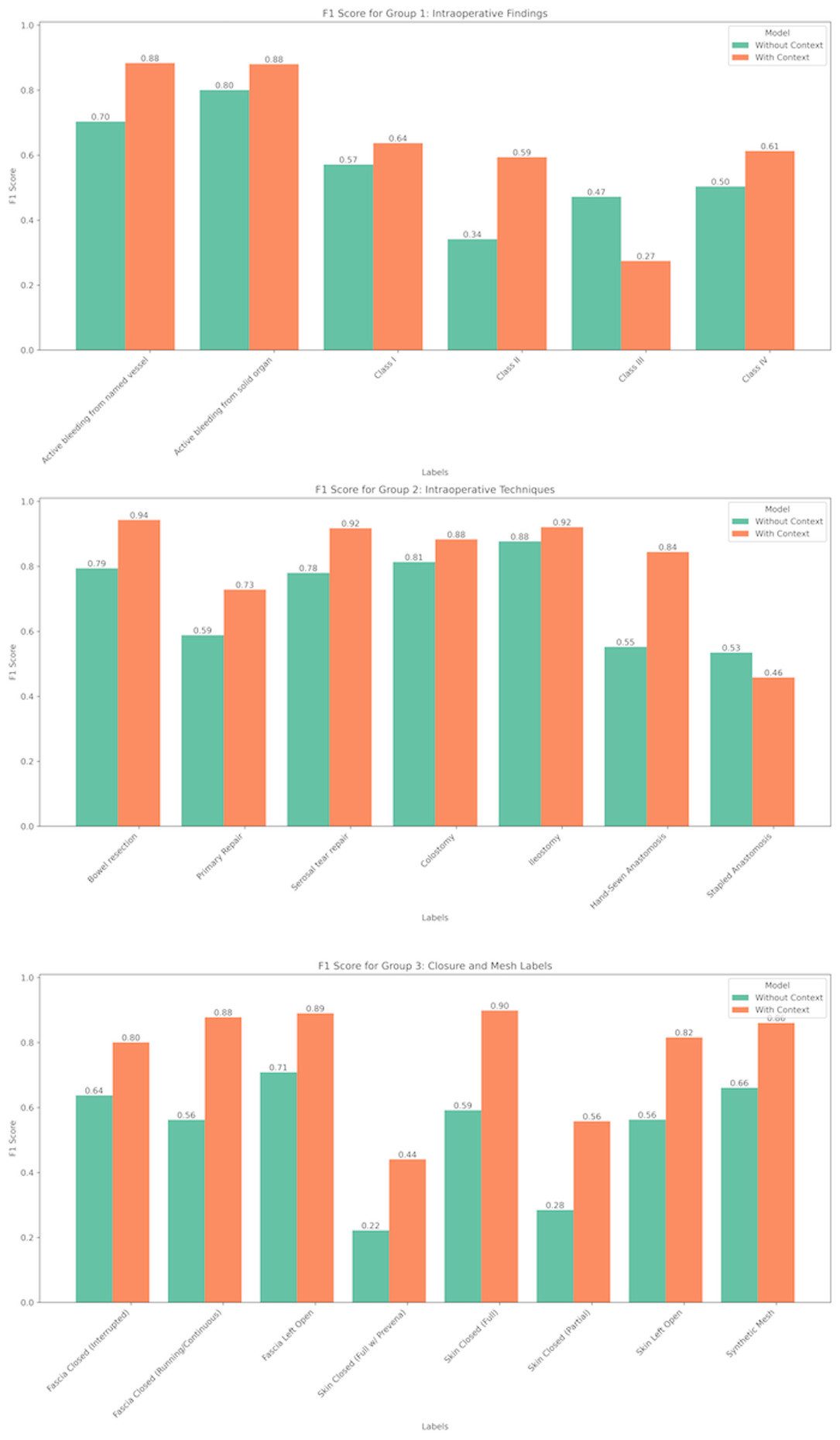

Supplement: Multimedia Appendix 6 [file medinform-v13-e71176-s006.docx]
